# Health-related quality of life among patients treated with lurasidone: results from a switch trial in patients with schizophrenia

**DOI:** 10.1186/1471-244X-14-53

**Published:** 2014-02-23

**Authors:** George Awad, Mariam Hassan, Antony Loebel, Jay Hsu, Andrei Pikalov, Krithika Rajagopalan

**Affiliations:** 1 Department of Psychiatry, University of Toronto, Toronto, ON, Canada; 2 Department of Psychiatry and Mental Health, Humber River Regional Hospital, Toronto, ON, Canada; 3 Sunovion Pharmaceuticals, Inc, Marlborough, MA, USA; 4 Sunovion Pharmaceuticals, Inc, Fort Lee, NJ, USA

**Keywords:** Health-related quality of life, Lurasidone, Antipsychotic, PETiT, SF-12

## Abstract

**Background:**

Patients with schizophrenia frequently switch between antipsychotics, underscoring the need to achieve and maintain important treatment outcomes such as health-related quality of life (HRQoL) following the switch. This analysis evaluated HRQoL changes among patients with schizophrenia switched from their current antipsychotic to lurasidone.

**Methods:**

Stable but symptomatic outpatients with schizophrenia were switched from their current antipsychotic to lurasidone in a six-week, open-label trial. HRQoL was assessed using two validated patient-reported measures, the Personal Evaluation of Transitions in Treatment (PETiT) scale and the Short-Form 12 (SF-12). Total and domain scores (psychosocial function and adherence-related attitude) were assessed using the PETiT scale; patients’ mental and physical component summary scores (MCS and PCS) were assessed using the SF-12. Changes in HRQoL from baseline to study endpoint were compared using ANCOVA, with baseline score, treatment, and pooled site as covariates. Changes were assessed among all patients and those switched from specific antipsychotics to lurasidone.

**Results:**

The analysis included 235 patients with data on the PETiT and SF-12 who had received ≥1 dose of lurasidone. Statistically significant improvements were observed from baseline to study endpoint on the PETiT total (mean change [SD]: 3.2 [8.5]) and psychosocial functioning (2.5 [6.9]) and adherence-related attitude (0.7 [2.6]) domain scores (all p ≤ 0.002). When examined by preswitch antipsychotic, significant improvements in PETiT total scores were observed in patients switched from quetiapine, risperidone, aripiprazole, and ziprasidone (all p < 0.03) but not olanzapine (p = 0.893). Improvements on the SF-12 MCS score were observed for all patients (mean change [SD]: 3.7 [11.5], p < 0.001) and for those switched from quetiapine or aripiprazole (both p < 0.03). The SF-12 PCS scores remained comparable to those at baseline in all patient groups.

**Conclusions:**

These findings indicate that patients switching from other antipsychotics to lurasidone experienced statistically significant improvement of HRQoL, based on PETiT scores, within six weeks of treatment. Patient health status remained stable with respect to the SF-12 physical component and showed improvement on the mental component. Changes in HRQoL varied based on the antipsychotic used before switching to lurasidone.

**Trial registration:**

NCT01143077.

## Background

Schizophrenia is a severe, chronic, and costly psychiatric disorder characterized by acute psychotic episodes. Affected individuals demonstrate a heterogeneous phenotype that includes a vast array of symptomology, variable responses to treatment, and poor health-related quality of life (HRQoL) [1-4]. Patients with schizophrenia can suffer from: (1) positive symptoms such as delusions, hallucinations, conceptual disorganization, suspiciousness, agitation, and hostility; and (2) negative symptoms such as blunted affect, emotional and social withdrawal, lack of spontaneity, and poverty of speech [5]. These disturbances have a pervasive impact on many areas of patient functioning and frequently reduce HRQoL. The cognitive deficits demonstrated by patients in the domains of executive function, attention, memory, and language are additionally recognized to negatively impact functional outcomes such as psychosocial functioning, work/education, and independent living [6-8]. Patient HRQoL may also be impacted directly by the treatments that are used to manage schizophrenia [9]. That is, while antipsychotic medications are likely to have a positive effect on patient well-being due to symptom improvements, differences in side effects among currently available therapies (e.g., rates of hyperprolactinemia, weight gain) may negatively impact functional status and overall HRQoL. The different drugs in the atypical antipsychotic class have varying pharmacological profiles, with differential impacts on the clinical response and adverse effects among patients; therefore, they can have a differential impact on HRQoL [10,11].

Patient adherence to treatment has also been considerably variable among different antipsychotics, and a patient’s subjective response or attitude to a therapy (i.e., how they perceive their clinical response and/or adverse effects) may impact adherence [12]. Since tolerability issues are common in the treatment of schizophrenia, patients often discontinue therapy or switch between different types of antipsychotic medications in an effort to find an optimal therapeutic regimen [13,14]. Moreover, patients with schizophrenia are often only partially adherent with their prescribed medications [15-17]. In a systematic review of 39 studies that assessed adherence using a variety of methods, approximately 40% of patients with the disorder were partially- or non-adherent to antipsychotic therapies [17]. While the specific cause is somewhat unclear, adherence-related attitude may play a role in poor adherence, potentially being associated with patient perceptions of medication efficacy and adverse effects [18-20].

Several studies have shown that poor adherence and/or treatment discontinuation are associated with an increased risk of relapse and re-hospitalization, both of which may negatively affect HRQoL [21-23]. Thus, high discontinuation and switching rates between antipsychotics underscores the need to ensure that important outcomes of treatment—such as enhanced adherence rates and improvements in HRQoL—are achieved and maintained following the switch to another antipsychotic.

Lurasidone is a second-generation atypical antipsychotic that received approval in October 2010 by the United States (US) Food and Drug administration (FDA) for the treatment of adult patients with schizophrenia [24]. Lurasidone can be differentiated from other available second-generation atypical antipsychotics by its receptor binding profile, with moderate affinities for the serotonin 5-HT7, noradrenaline α2c (antagonist), and serotonin 5-HT1A (weak-moderate partial agonist), in addition to the expected high affinity binding for dopamine D2 and serotonin 5-HT2A receptors. Lurasidone has little to no appreciable affinity for the 5-HT2C, histamine H1, and acetylcholine M1 receptors.

The results of a recently published study demonstrated that switching clinically stable yet symptomatic patients with schizophrenia or schizoaffective disorder to lurasidone from other antipsychotic agents was well tolerated, with low rates of patient discontinuation [25]. This analysis aimed to assess changes in HRQoL in patients with schizophrenia who were switched to lurasidone from other antipsychotic agents in a six-week open-label multicenter parallel group trial using the Personal Evaluation of Transitions in Treatment (PETiT) scale. In addition to overall HRQoL, the study evaluated changes in several important domains of HRQoL in schizophrenia (adherence-related attitude, psychosocial functioning, social functioning, activity, patient perception of cognition, and dysphoria) as measured by PETiT domain scores. The secondary objective of the analysis included an assessment of general health status in patients switching to lurasidone using the Short-Form 12 (SF-12).

## Methods

### Core study design

The analysis was based on data from a six-week, open-label, parallel-group trial of stable but symptomatic outpatients with schizophrenia who were switched from their current antipsychotic to lurasidone [25]. The detailed methodology of this study has been reported previously [25]. Briefly, the study was conducted at 28 sites in the United States (ClinicalTrials.gov identifier: NCT01143077). The study protocol was reviewed and approved by an institutional review board at each study center, and the trial was conducted in accordance with Good Clinical Practice as required by the International Conference on Harmonization guidelines. Compliance with these requirements also constitutes conformity with the ethical principles of the Declaration of Helsinki. Subjects had to provide informed consent to participate in the study. Eligible subjects were adults with clinically stable, Diagnostic and Statistical Manual of Mental Disorders IV (DSM-IV)–defined schizophrenia or schizoaffective disorder who were considered appropriate candidates for switching from their current antipsychotic medications (due to insufficient efficacy and/or safety or tolerability concerns). Subjects were randomized to one of three lurasidone dosing regimens for the initial two weeks of the study: (1) 40 mg/d for two weeks; (2) 40 mg/d for one week, then increased to 80 mg/d for week two; and (3) 80 mg/d for two weeks. Over the initial two week course, the preswitch antipsychotic was tapered to 50% at the first week visit and discontinued totally at the second week visit. Lurasidone was then flexibly dosed (40–120 mg/d) for the subsequent four weeks. Patients randomized to all three dosing regimens of lurasidone were pooled together for the study analysis. The core clinical trial categorized subjects switched from olanzapine or quetiapine into the sedating antipsychotic group and patients switched from risperidone, aripiprazole, or ziprasidone into the non-sedating antipsychotic group a priori to the study [25]. This categorization was an assumption based on literature suggesting that olanzapine and quetiapine have greater sedating characteristics than risperidone, aripiprazole, and ziprasidone [26,27].

The primary study outcome was time to treatment failure, defined as any occurrence of insufficient clinical response, exacerbation of underlying disease, or discontinuation due to an adverse event (AE), as determined by investigator judgement. HRQoL and general health status, including evaluation of physical functioning and mental health, were studied as secondary endpoints using the PETiT and SF-12 Patient Reported Outcomes measures. The PETiT and SF-12 assessments were administered at baseline and at six weeks.

### Outcome measures

#### ***(i) PETiT Scale***

The PETiT scale is a validated, 30-item instrument designed to capture and quantify the impact of treatment on self-perceived subjective aspects of patient HRQoL [28]. The scale is known to assess two relevant domains: 1) adherence-related attitude (six items, including adherence and feelings towards medication) and psychosocial functioning (24 items, including clarity, energy, concentration, functioning, sex drive, and memory). Psychosocial functioning was further assessed in terms of four sub-domains: social functioning (four items on trust, confidence, and interactions), activity (seven items on energy, ability to conduct daily tasks), cognitive (seven items on clarity, concentration, and communication), and dysphoria (six items on happiness, future, and self-esteem). Each item of the PETiT scale is assigned a rating of 0, 1, or 2, where 0 denotes a negative change (i.e., worse HRQoL) and 2 denotes a positive change (i.e., better HRQoL). Total PETiT scale score ranges from 0 to 60, with higher scores on PETiT denoting better HRQoL.

#### ***(ii) SF-12***

Quality of life outcomes were also assessed in patients switched to lurasidone using the SF-12 survey, a multipurpose generic measure of health status [29]. The SF-12 yields scale scores for items such as physical functioning, role limitations, health perceptions, bodily pain, vitality, social functioning, and mental health on the basis of patient responses to 12 questions. The survey yields two summary measures of physical and mental health: the Physical Component Summary (PCS) and the Mental Component Summary (MCS).

#### ***Analysis***

The intent-to-treat (ITT) population was used for the PETiT and SF-12 analysis. The ITT population was defined as all patients who had received at least one dose of lurasidone and had non-missing values for PETiT and SF-12 scores at baseline and ≥1 post-baseline value at study endpoint. The study endpoint was the last observation carried forward (LOCF), defined as the last non-missing value for any PETiT or SF-12 item at a scheduled or unscheduled visit post-baseline. Mean changes from baseline to LOCF in PETiT and SF-12 scores were calculated using analysis of covariance (ANCOVA) models, with treatment and pooled center as fixed factors and baseline value as a covariate.

Mean changes from baseline to LOCF for the PETiT scale total score, its domains, and the SF-12 PCS and MCS scores were determined for all patients in the ITT population. The analysis further examined PETiT and SF-12 scores by the individual preswitch antipsychotic medications that were received by ≥10% of patients in the study. Scores were additionally examined by categorizing these medications into the sedating (olanzapine and quetiapine) and non-sedating (risperidone, aripiprazole, and ziprasidone) subgroups. Finally, the analysis also examined HRQoL among patients who had completed or discontinued treatment with lurasidone due to any cause at study endpoint.

## Results

### Patient demographics & baseline characteristics

The study population was comprised of 240 patients with schizophrenia or schizoaffective disorder who received at least one dose of study medication. Table 1 presents the baseline clinical characteristics for the total study population. Of the 240 patients switched to lurasidone from other antipsychotics, 235 patients with available data on the PETiT scale and SF-12 assessment comprised the ITT population in the current analysis. The majority of patients were male (65%) and the mean age at study entry was 43.9 years. For the purpose of this study, 152 of 235 patients (65%) were treated with a preswitch non-sedating antipsychotic (risperidone, aripiprazole, ziprasidone) and 83 of 235 (35%) were treated with a preswitch sedating medication (olanzapine or quetiapine).

**Table 1:** Patient demographics and baseline clinical characteristics

**Parameter**	No. of subjects (%)*
N	240
Mean age	
Years, SD	43.9 (10.9)
Gender	
Male	156 (65.0%)
Female	84 (35.0%)
Race	
Asian	1 (0.4%)
Black or African American	151 (62.9%)
Native Hawaiian or other Pacific Islander	1 (0.4%)
White	80 (33.3%)
Other	7 (2.9%)
DSM-IV Schizophrenia subtype diagnosis	
295.10 Disorganized type	4 (1.7%)
295.20 Catatonic type	0
295.30 Paranoid type	125 (52.1%)
295.60 Residual type	2 (0.8%)
295.70 Schizoaffective disorder	89 (37.1%)
295.90 Undifferentiated type	21 (8.8%)
Preswitch antipsychotic agent at study start	
Quetiapine	62 (25.8%)
Risperidone	51 (21.3%)
Aripiprazole	44 (18.3%)
Ziprasidone	27 (11.3%)
Olanzapine	24 (10.0%)
Paliperidone	9 (3.8%)
Iloperidone	4 (1.7%)
Asenapine	2 (0.8%)
First-generation antipsychotic	17 (7.1%)
Treatment with concomitant lithium, valproate or lamotrigine	34 (16.2%)
Treatment with concomitant antidepressant	104 (43.3%)
Mean age (SD) at initial onset of schizophrenia or schizoaffective disorder, years	25.1 (9.3)
Mean positive and negative syndrome scale total score (SD)	68.9 (13.8)
Mean clinical global impression severity score (SD)	3.7 (0.5)

*or as indicated.

### PETiT assessment

The mean (± standard deviation [SD]) PETiT total score for all lurasidone patients improved from 35.0 (8.8) at baseline to 38.5 (9.2) at LOCF endpoint, representing a mean improvement of 3.2 (8.5) or 9.1% (p < 0.001). Improvements from baseline to LOCF endpoint in the total score, as well as in the domains of adherence-related attitude (0.7 [2.6]) and psychosocial functioning (2.5 [6.9]), were statistically significant (p ≤ 0.002) for all patients who were switched to lurasidone (Table 2). All aspects of the psychosocial functioning domain (activity, cognitive, and dysphoria) showed significant improvement (p ≤ 0.002) with the exception of social functioning, where a non-significant improvement was demonstrated.

**Table 2:** Mean change in PETiT assessments among patients switched to lurasidone

	**Parameter**	All patients*	**Sedating**	**Non-sedating**
		**(N = 235)**	**(n = 83)**	**(n = 152)**
PETiT total score	Baseline (SD)	35.0 (8.8)	33.8 (8.6)	35.7 (8.9)
	LOCF (SD)	38.5 (9.2)	36.5 (10.1)	39.6 (8.5)
	Mean change (SD)	3.2 (8.5)	2.7 (9.3)	3.5 (8.1)
	p-value	< 0.001	0.101	< 0.001
Adherence-related attitude domain score (6 items)	Baseline (SD)	8.7 (2.1)	8.4 (2.0)	8.8 (2.1)
	LOCF (SD)	9.4 (2.2)	8.9 (2.6)	9.7 (2.0)
	Mean change (SD)	0.7 (2.6)	0.5 (2.8)	0.8 (2.4)
	p-value	0.002	0.735	< 0.001
Psychosocial functioning domain score (24 items)	Baseline (SD)	26.4 (7.7)	25.4 (7.6)	26.9 (7.8)
	LOCF (SD)	29.1 (7.9)	27.7 (8.6)	29.9 (7.4)
	Mean change (SD)	2.5 (6.9)	2.1 (7.4)	2.7 (6.6)
	p-value	< 0.001	0.074	< 0.001
Social functioning (4 items)	Baseline (SD)	3.9 (1.4)	3.6 (1.4)	4.0 (1.4)
	LOCF (SD)	4.0 (1.5)	3.6 (1.5)	4.2 (1.5)
	Mean change (SD)	0.1 (1.4)	-0.1 (1.5)	0.1 (1.4)
	p-value	0.959	0.066	0.198
Activity (7 items)	Baseline (SD)	7.7 (2.8)	7.6 (2.8)	7.8 (2.8)
	LOCF (SD)	8.5 (2.9)	8.3 (3.0)	8.6 (2.9)
	Mean change (SD)	0.7 (2.7)	0.6 (2.8)	0.8 (2.7)
	p-value	0.002	0.124	0.002
Cognitive (7 items)	Baseline (SD)	8.1 (2.8)	7.8 (2.7)	8.3 (2.8)
	LOCF (SD)	9.1 (2.6)	8.8 (2.9)	9.3 (2.5)
	Mean change (SD)	0.9 (2.5)	0.9 (2.8)	0.9 (2.4)
	p-value	< 0.001	0.006	< 0.001
Dysphoria (6 items)	Baseline (SD)	6.7 (2.5)	6.4 (2.3)	6.8 (2.6)
	LOCF (SD)	7.5 (2.4)	7.0 (2.7)	7.8 (2.1)
	Mean change (SD)	0.8 (2.3)	0.7 (2.4)	0.9 (2.2)
	p-value	< 0.001	0.149	< 0.001

*Patients eligible for evaluation in the analysis (N = 235) may have non-missing values at baseline and ≥1 post-baseline value at study endpoint (LOCF) for any PETiT items; n values may not sum to 235 due to missing data.

Note: preswitch sedating medications include quetiapine and olanzapine; preswitch non-sedating medications include risperidone, aripiprazole, and ziprasidone.

#### ***PETiT scores by preswitch antipsychotic medication***

The differences in patients’ PETiT scores were also stratified based on the antipsychotic medication used prior to switching to lurasidone. To ensure a reasonable sample size for this analysis, preswitch antipsychotic medications received by ≥10% of patients in the study were included for stratification. The medications included quetiapine (n = 62), risperidone (n = 51), aripiprazole (n = 44), ziprasidone (n = 27), and olanzapine (n = 24). Patients on all of these preswitch medications except olanzapine showed statistically significant improvements in total PETiT scores, as determined by mean changes from baseline to LOCF (±SD): quetiapine 4.2 (7.7), p = 0.011; risperidone 3.6 (7.9), p = 0.029; aripiprazole 3.4 (8.0), p = 0.010; ziprasidone 5.4 (7.9), p = 0.009 (Table 3). Patients on these four agents also showed significant improvements on the psychosocial functioning component (all p < 0.05) (Table 3). For patients switched from olanzapine, a numerical decrease in the total PETiT score and its components was observed; however, this difference was not statistically significant. Patients in the aripiprazole and ziprasidone preswitch groups additionally showed statistically significant improvements in the adherence-related attitude component (both p < 0.05).

**Table 3:** Mean change in PETiT assessments by preswitch medication among patients switched to lurasidone (N = 235)*

	**Parameter**	**Quetiapine**	**Olanzapine**	**Risperidone**	**Aripiprazole**	**Ziprasidone**
		**(n = 62)**	**(n = 24)**	**(n = 51)**	**(n = 44)**	**(n = 27)**
PETiT total score	Baseline (SD)	31.6 (7.8)	39.1 (9.9)	38.3 (8.7)	35.1 (6.9)	34.0 (8.5)
	LOCF (SD)	36.1 (8.5)	37.5 (13.8)	41.6 (8.2)	38.7 (9.1)	39.3 (7.6)
	Mean change (SD)	4.2 (7.7)	-1.3 (11.8)	3.6 (7.9)	3.4 (8.0)	5.4 (7.9)
	p-value	0.011	0.893	0.029	0.010	0.009
Adherence-related attitude domain score (6 items)	Baseline (SD)	8.0 (1.9)	9.1 (2.1)	9.2 (2.1)	8.4 (2.0)	8.6 (2.0)
	LOCF (SD)	8.8 (2.3)	9.1 (3.0)	9.9 (2.1)	9.5 (2.2)	9.8 (1.9)
	Mean change (SD)	0.8 (2.4)	-0.4 (3.4)	0.8 (2.0)	1.0 (2.9)	1.2 (2.0)
	p-value	0.150	0.871	0.060	0.026	0.046
Psychosocial functioning domain score (24 items)	Baseline (SD)	23.6 (6.9)	30.1 (8.8)	29.2 (7.6)	26.8 (6.4)	25.4 (7.3)
	LOCF (SD)	27.3 (7.5)	28.4 (11.2)	31.7 (7.3)	29.2 (7.7)	29.5 (6.7)
	Mean change (SD)	3.4 (6.3)	-1.1 (9.1)	2.8 (7.0)	2.3 (6.1)	4.2 (6.6)
	p-value	0.015	0.898	0.048	0.020	0.006

*Patients eligible for evaluation in the analysis (N = 235) may have non-missing values at baseline and ≥1 post-baseline value at study endpoint (LOCF) for any PETiT items; n values may not sum to 235 due to missing data.

#### ***PETiT scores by patients switched from sedating and non-sedating antipsychotics***

Differences in PETiT scores were also found between patients who had received a non-sedating antipsychotic (risperidone, aripiprazole, ziprasidone) and those who had received a sedating antipsychotic (olanzapine or quetiapine) prior to the switch to lurasidone. In the non-sedating group, statistically significant (p < 0.001) improvements from baseline to LOCF endpoint were observed for the total PETiT score and its psychosocial functioning and adherence-related attitude domains (Table 2). While numerical improvements in the scores for these three outcomes were observed in the sedating group, these changes were not statistically significant.

#### ***PETiT scores by study discontinuation status***

Patients were categorized as subjects who discontinued (37 [16%]) or subjects who completed (198 [84%]) with lurasidone in the ITT population based on discontinuation due to any cause at the six-week endpoint. When analyzed by discontinuation status, the study showed that patients who completed treatment with lurasidone had significantly improved PETiT total scores versus patients who discontinued treatment (p < 0.001) (Table 4). This improvement was also observed in the adherence-related attitude and psychosocial functioning domains of the PETiT scale (both p < 0.001).

**Table 4:** Mean change in PETiT assessments by discontinuation status among patients switched to lurasidone

	**Parameter**	**All patients**		**Sedating**		**Non-sedating**	
		Discontinued*	**Completed**	Discontinued*	**Completed**	Discontinued*	**Completed**
		**(n = 37)**	**(n = 198)**	**(n = 18)**	**(n = 65)**	**(n = 19)**	**(n = 133)**
PETiT total score	Baseline (SD)	34.3 (7.6)	35.1 (9.0)	33.2 (6.9)	33.9 (9.0)	35.4 (8.3)	35.7 (9.0)
	LOCF (SD)	33.7 (11.0)	39.1 (8.9)	30.9 (11.5)	37.5 (9.6)	36.8 (10.0)	39.8 (8.4)
	Mean change (SD)	-3.6 (12.4)	4.0 (7.7)	-4.5 (14.7)	3.9 (7.5)	-2.6 (9.9)	4.0 (7.8)
	p-value**	< 0.001		0.008		0.004	
Adherence-related attitude domain score (6 items)	Baseline (SD)	8.7 (1.7)	8.7 (2.1)	8.6 (1.2)	8.3 (2.2)	8.7 (2.2)	8.8 (2.1)
	LOCF (SD)	8.0 (3.4)	9.6 (2.0)	7.4 (3.7)	9.1 (2.2)	8.6 (3.2)	9.8 (1.9)
	Mean change (SD)	-1.4 (3.4)	0.9 (2.4)	-1.6 (4.2)	0.8 (2.3)	-1.1 (2.6)	1.0 (2.4)
	p-value**		< 0.001		0.003		0.010
Psychosocial functioning domain score (24 items)	Baseline (SD)	25.6 (6.9)	26. 5 (7.9)	24.6 (6.5)	25.6 (7.9)	26.6 (7.2)	26.9 (7.9)
	LOCF (SD)	25.8 (8.2)	29.5 (7.8)	23.5 (8.5)	28.4 (8.4)	28.2 (7.4)	30.0 (7.5)
	Mean change (SD)	-2.2 (9.7)	3.0 (6.3)	-2.9 (11.3)	3.0 (6.2)	-1.5 (8.2)	3.1 (6.4)
	p-value**	< 0.001		0.028		0.011	

*Subjects who discontinued treatment with lurasidone due to any reason.

**Comparison of mean change between subjects who discontinued versus completed treatment with lurasidone at 6-week endpoint.

Note: preswitch sedating medications include quetiapine and olanzapine; preswitch non-sedating medications include risperidone, aripiprazole, and ziprasidone.

### SF-12 assessment

For all patients, the results of the SF-12 revealed that health status remained stable following the switch to lurasidone, with small improvements observed for both the PCS and MCS scores (Table 5). Improvements on the MCS score were noted in all subgroups (all patients, sedating, and non-sedating groups) following the switch to lurasidone, with statistically significant differences observed in the all patients (mean [SD]: 3.7 [11.5], p < 0.001) and non-sedating (3.7 [10.4], p < 0.001) subgroups. Overall, analysis of patients by preswitch antipsychotic agent revealed little difference between baseline and LOCF scores for most medications (olanzapine, risperidone, ziprasidone); however, significant increases in MCS scores were noted for the patients switched from quetiapine (4.2 [11.3], p = 0.029) and aripiprazole (4.7 [10.4], p = 0.002) (Table 6). Although not statistically significant, the increase in MCS score (5.6 [10.2]) in patients switched from ziprasidone was considered clinically significant (i.e., a change in score of ±5).

**Table 5:** Mean change in SF-12 physical and mental component summary scores among patients switched to lurasidone

	**Parameter**	**All patients**	**Sedating**	**Non-sedating**
		(N = 235)*	**(n = 83)**	**(n = 152)**
Physical component summary	Baseline (SD)	47.1 (10.1)	47.1 (10.4)	47.1 (10.0)
	LOCF (SD)	47.0 (9.8)	46.8 (9.6)	47.1 (9.9)
	Mean change (SD)	-0.2 (8.5)	-0.3 (8.2)	-0.2 (8.7)
	p-value	0.414	0.513	0.556
Mental component summary	Baseline (SD)	41.4 (11.4)	40.1 (11.6)	42.1 (11.2)
	LOCF (SD)	45.2 (11.1)	44.2 (12.5)	45.8 (10.2)
	Mean change (SD)	3.7 (11.5)	3.7 (13.3)	3.7 (10.4)
	p-value	< 0.001	0.079	< 0.001

*Patients eligible for evaluation in the analysis (N = 235) had non-missing values at baseline and ≥1 post-baseline value at study endpoint (LOCF) for any SF-12 items; n values may not sum to 235 due to missing data.

Note: preswitch sedating medications include quetiapine and olanzapine; preswitch non-sedating medications include risperidone, aripiprazole, and ziprasidone.

**Table 6:** Mean changes in SF-12 physical and mental component summary scores by preswitch medication* among patients switched to lurasidone

	**Parameter**	**Quetiapine**	**Olanzapine**	**Risperidone**	**Aripiprazole**	**Ziprasidone**
		**(n = 62)**	**(n = 24)**	**(n = 51)**	**(n = 44)**	**(n = 27)**
Physical component summary	Baseline (SD)	45.8 (10.3)	50.1 (9.5)	48.1 (8.5)	46.9 (11.0)	48.6 (10.5)
	LOCF (SD)	44.1 (9.6)	51.0 (8.8)	50.4 (8.8)	46.0 (10.1)	47.0 (9.9)
	Mean change (SD)	-1.3 (9.0)	1.3 (6.0)	2.4 (9.1)	-2.1 (7.9)	-0.4 (6.8)
	p-value	0.046	0.077	0.124	0.190	0.427
Mental component summary	Baseline (SD)	38.9 (10.9)	43.8 (12.8)	43.8 (10.9)	42.2 (9.8)	39.5 (10.0)
	LOCF (SD)	44.2 (10.9)	44.3 (15.7)	46.2 (10.0)	45.1 (9.2)	44.9 (10.4)
	Mean change (SD)	4.2 (11.3)	0.0 (15.0)	2.6 (10.8)	4.7 (10.4)	5.6 (10.2)
	p-value	0.029	0.834	0.298	0.002	0.129

*Patients eligible for evaluation in the analysis (N = 235) may have had non-missing values at baseline and ≥1 post-baseline value at study endpoint (LOCF) for any SF-12 items; n values may not sum to 235.

When analyzed by discontinuation status, a statistically significant improvement in the MCS score was observed among patients who remained on lurasidone in the all patients (p = 0.029) and sedating subgroups (p = 0.036) versus those who had discontinued treatment at the six-week endpoint (Table 7). No difference was noted in the PCS and MCS scores of patients switching from non-sedating antipsychotics.

**Table 7:** Mean changes in SF-12 physical and mental component summary scores by discontinuation status among patients switched to lurasidone

	**Parameter**	**All patients**		**Sedating**		**Non-sedating**	
		Discontinued*	**Completed**	Discontinued*	**Completed**	Discontinued*	**Completed**
		**(n = 37)**	**(n = 198)**	**(n = 18)**	**(n = 65)**	**(n = 19)**	**(n = 133)**
Physical component summary	Baseline (SD)	46.8 (8.8)	47.2 (10.4)	48.3 (9.0)	46.8 (10.7)	45.3 (8.6)	47.3 (10.2)
	LOCF (SD)	46.6 (10.2)	47.0 (9.8)	50.6 (7.2)	46.1 (9.9)	42.3 (11.6)	47.4 (9.8)
	Mean change (SD)	-1.1 (9.6)	-0.1 (8.4)	1.5 (5.7)	-0.6 (8.5)	-3.9 (12.3)	-0.1 (8.3)
	p-value**	0.915		0.142		0.106	
Mental component summary	Baseline (SD)	41.7 (11.4)	41.3 (11.4)	39.9 (11.9)	40.2 (11.7)	43.3 (11.0)	41.9 (11.3)
	LOCF (SD)	42.3 (12.2)	45.5 (10.9)	38.8 (14.7)	45.1 (12.0)	46.1 (7.5)	45.8 (10.4)
	Mean change (SD)	-1.6 (14.6)	4.3 (11.0)	-3.5 (18.9)	4.9 (11.9)	0.5 (8.4)	4.0 (10.5)
	p-value**	0.029		0.036		0.498	

*Subjects who discontinued treatment with lurasidone due to any reason, at 6-week endpoint.

**Comparison of mean change between subjects who discontinued versus completed treatment with lurasidone at 6-week endpoint.

Note: preswitch sedating medications include quetiapine and olanzapine; preswitch non-sedating medications include risperidone, aripiprazole, and ziprasidone.

## Discussion

Along with efficacy and safety, maintenance or improvement of HRQoL is an important outcome of treatment for patients with schizophrenia. This study is the first to systematically examine the effects of switching clinically stable patients with schizophrenia from their current antipsychotic to lurasidone on HRQoL.

The PETiT scale offers a specific and integrated measure of HRQoL for patients who have switched antipsychotic medications, where a patient’s well-being is conceptualized as their subjective perception of their severity of psychotic symptoms, medication side effects, and level of psychosocial performance [28]. The SF-12 offers a more generic and well-recognized evaluation of physical and mental status that permits comparison to outcomes with other disorders. In populations such as that included in the lurasidone switch study, where patients were clinically stable yet symptomatic at baseline, it would be expected that switching to a new medication might lead to only marginal improvements in terms of these HRQoL outcomes. Therefore, the statistically significant improvements demonstrated by the PETiT assessment after only six weeks of lurasidone therapy are notable and clinically important for patients switching from other antipsychotics.

The majority of patients in the switch study showed improvements from baseline to LOCF on the PETiT total score and the domains of adherence-related attitude, psychosocial functioning, activity, patient perception of cognition, and dysphoria. These findings indicate that, in this study, patients switching to lurasidone perceived improvements in a broad range of measures of well-being. The finding of improved adherence-related attitude following switch to lurasidone is of particular importance, considering the role of patient perception (e.g., of medication, clinical efficacy, AEs) in the traditionally high rates of non-adherence and discontinuation associated with antipsychotic medications [15-17] and the potential cost and HRQoL implications of inadequate treatment (e.g., due to psychotic relapse, hospitalization) [21,30]. The higher PETiT scores observed among patients who completed lurasidone treatment provides evidence that patient-reported HRQoL may be associated with the likelihood of continuing treatment.

When examined by preswitch antipsychotic, changes in HRQoL were more variable. Patients switched from quetiapine, risperidone, aripiprazole, and ziprasidone showed statistically significant improvements in PETiT total scores. However, there were no significant changes in PETiT scores among patients switched from olanzapine. It is known that drugs in the atypical antipsychotic class differ in pharmacological profiles, clinical response, and the adverse effects experienced by patients [10,11]. Measures of HRQoL allow patients to consider both their clinical response and adverse effects and to emphasize the treatment effect that is of greater relevance to them. In this study, the improvements in HRQoL that were observed after switching to lurasidone from widely-used antipsychotic agents with variable adverse-effect profiles (quetiapine, risperidone, aripiprazole, and ziprasidone), and the maintenance of HRQoL after switching from the highly efficacious antipsychotic olanzapine, collectively suggest that lurasidone is both effective and well tolerated.

The PETiT analysis additionally showed differences in HRQoL depending on whether the pre-study medication was sedating or non-sedating. Patients switching from non-sedating medications showed statistically significant improvements in the total, adherence-related attitude, and psychosocial functioning scores of the PETiT scale; in contrast, the improvements observed in the sedating group were not statistically significant. The difficulty in switching patients from sedating to non-sedating atypical antipsychotics is a well-known challenge in the treatment of schizophrenia [31]. Subjective tolerability—how a patient feels on their medication—may play a role in this challenge, potentially contributing to the greater improvements on the PETiT score in patients switching from non-sedating versus sedating antipsychotics [22,32,33]. Results published earlier from this study also revealed differences in the time to treatment discontinuation and all-cause discontinuation between patients switched from sedating versus non-sedating antipsychotic agents [25]. The authors suggested that attention should be paid to the emergence of insomnia or anxiety in persons who had received a sedating antipsychotic immediately prior to switching to lurasidone.

Finally, the results of the more generic SF-12 assessment also support the feasibility of switching to lurasidone from other antipsychotics. Patients generally demonstrated little change or improvements in the PCS and MCS scores, indicating that their physical and mental health status was maintained or improved by switching to lurasidone. Given the clinical stability of the patient population at baseline and the short six-week duration of follow-up, it is not unexpected that no marked difference was observed in physical component using a generic instrument such as the SF-12 [34].

Overall, it is well recognized that the HRQoL of patients with schizophrenia can be negatively impacted by the effects of atypical antipsychotic therapies [9-11]. The findings of the current analysis are therefore important, as maintenance or improvement of patient well-being following switch to lurasidone may in turn make patients more likely to adhere to and continue on therapy. As noted previously, improvements in adherence and continuation of treatment may improve patient outcomes, such as reductions in relapse and re-hospitalization events [23,30].

This analysis is one of few published studies to examine changes in HRQoL, functioning, and health status after switching between antipsychotics. While four relatively recent investigations of patients switching to quetiapine XR [35], aripiprazole [36], ziprasidone [37], or long-acting injectable risperidone [38] reported on changes in cognitive function, psychotic symptoms, and tolerability, only one additionally described changes in quality of life using the Subjective Well-being Under Neuroleptics Scale – Brief Form [SWN-K] [37]. This study reported no significant change in patient quality of life following switch to aripiprazole [37].

Other studies have commented on the risk of tolerability problems, symptom exacerbations, or increased use of acute care services after switching patients between antipsychotics [39-41]. However, the results of this clinical trial, as reported by McEvoy and colleagues [25] and described herein, demonstrate that switching to lurasidone has a low risk of treatment failure, discontinuation, AEs, or of an adverse impact on patient well-being.

There are a few limitations of the current study. First, being an open-label evaluation with no control group, the outcomes were prone to greater bias than outcomes from a randomized controlled clinical trial. Notwithstanding this limitation, this naturalistic switch trial has potential application for clinical practice guidance on switching patients to lurasidone. Second, the six-week duration of the study may not have been long enough to fully capture changes in HRQoL and other outcomes. However, such outcomes remain a critical source of insight concerning numerous aspects of any disease, and in particular, the perception of patient well-being in psychiatric disorders such as schizophrenia. Analysis of the longer-term effect of lurasidone on HRQoL, in both the PETiT and SF-12 assessments, from the six-month extension phase of the trial is ongoing. Another limitation was the study’s small sample size for the subgroup analyses, and interpretation of the subgroup results warrants caution. Finally, as noted previously by McEvoy and colleagues [25], the lack of information on the preswitch sedation status of patients is a limiting factor in terms of understanding the validity of categorizing the preswitch agents as “sedating” or “non-sedating”. Still, the clinical and now quality of life outcomes observed in this study suggest that this distinction may be clinically relevant to patients with schizophrenia. As suggested by McEvoy’s group, stratification of the data on the basis of agent or properties other than sedation could result in different outcomes than those reported here.

Despite these limitations, the study results suggest that stable yet symptomatic patients with schizophrenia may be efficiently switched from other antipsychotics to lurasidone, with potential improvements in psychosocial functional, attitude related to adherence, and overall mental health status.

## Conclusions

In conclusion, the results of the PETiT and SF-12 assessments indicate that patients with schizophrenia who switch to lurasidone from other antipsychotics may experience improvements in HRQoL within six weeks of treatment. Further investigation of the effects of longer-term lurasidone therapy on quality of life outcomes and patient-reported perception of switching to lurasidone is warranted.

## Abbreviations

AE: Adverse events; ANCOVA: Analysis of covariance; DAI: Drug attitude inventory; DSM: Diagnostic and statistical manual of mental disorders; FDA: Food and drug administration; HRQoL: Health-related quality of life; ITT: Intention to treat; LOCF: Last observation carried forward; MCS: Mental component score; PETiT: Personal evaluation of transitions in treatment; PCS: Physical component score; SF-12: Short-form 12; US: United states; SD: Standard deviation; SWN-K: Subjective well-being under neuroleptics scale – brief form.

## Competing interests

Mariam Hassan, Antony Loebel, Jay Hsu, Andrei Pikalov, and Krithika Rajagopalan are employees of Sunovion Pharmaceuticals, Inc. George Awad has no competing interests to declare.

## Authors’ contributions

MH and KR conceptualized the post-hoc analysis. GA, MH, KR, AL, AP and JH participated in the study analysis and data interpretation. AL, AP, and JH were involved in the conceptualizing, designing and analysis of the clinical trial. GA, MH, KR, AP, and AL critically reviewed and revised the manuscript for important intellectual content. All authors have reviewed and approved this manuscript.

## Pre-publication history

The pre-publication history for this paper can be accessed here:

http://www.biomedcentral.com/1471-244X/14/53/prepub
